# TREK-1 and epilepsy: regulating the balance of K^+^ and the glutamate release in astrocyte-neuron interactions

**DOI:** 10.1186/s12993-025-00294-x

**Published:** 2025-08-31

**Authors:** Jianing Yang, Li Li, Yanan Xu, Yuguang Guan, Xiaoli Li

**Affiliations:** 1 Department of Neurology, Affiliated ZhongDa Hospital, Southeast University, Nanjing, China; 2 Medical College, Southeast University, Nanjing, China; 3 Health Management Center, Affiliated ZhongDa Hospital, Southeast University, Nanjing, China; 4 Department of Neurosurgery, SanBo Brain Hospital, Capital Medical University, Beijing, China

**Keywords:** Astrocyte, Epilepsy, Glutamate, Potassium channel, TREK-1

## Abstract

The TWIK-related K^+^ channel (TREK-1), a member of the two-pore domain potassium(K2P) family, is characterized as a “leaky potassium channel” and is integral to the maintenance of the resting membrane potential. As the most abundant cell type in the central nervous system, astrocytes play important roles in the development of epilepsy by regulating the release of glutamate and the function of potassium channels. Previous studies have revealed that TREK-1 is involved in a range of neurological diseases, including epilepsy. In astrocytes, TREK-1 acts as a crucial regulator of the rapid release of glutamate and passive conductance. However, controversy remains about the expression levels of TREK-1-binding receptors in the process of the release and recycling of glutamate in tripartite synapses. Thus, elucidating the pathological mechanisms involving TREK-1 in epilepsy could significantly increase our understanding of the pathophysiological basis of diseases and facilitate the identification of potential targets for novel therapeutic interventions. Here, we review the physiological function of TREK-1 and studies examining the role of TREK-1 in epilepsy, with a particular emphasis on its interactions with glutamate at tripartite synapses. Furthermore, we provide an analysis of the associated molecular mechanisms of this channel and conclude with an outlook on impending studies on TREK-1 as a novel therapeutic target for epilepsy.

## Introduction

Approximately 70 million individuals worldwide are affected by epilepsy, one of the most prevalent neurological disorders [1]. This condition is attributed primarily to abnormal discharges of synchronized neurons within the brain. The evidence for the predominant hypotheses of epilepsy points to a glutamate imbalance and potassium channel dysfunction [2].

The TWIK-related K^+^ channel (TREK-1), the most extensively studied member of the two-pore domain potassium (K2P) channel family, is widely expressed in various tissues, particularly within the central nervous system (CNS); its unique background of leakage conductance is crucial for maintaining the resting membrane potential and neuronal excitability [3]. Moreover, TREK-1 is regulated by a diverse array of physical and chemical stimuli, including pH, temperature, mechanical tension, polyunsaturated fatty acids, and neurotransmitters [4–8]. Numerous studies have implicated TREK-1 in the pathophysiology of neurological disorders, such as epilepsy, depression, ischaemia, and pain [9–12]. Additionally, TREK-1 is regarded as a key target for anaesthetic agents [13]; the channel is hypothesized to play a role in both of the aforementioned mechanisms in epilepsy, which are believed to interact with one another [14]. Moreover, TREK-1 has been found to be involved in the development of the comorbidities of epilepsy, such as depression and cognition disorders [15, 16].

There is increasing interest in the role of astrocyte-neuron interactions in epileptogenesis [17]. The key structure involved in the interaction between astrocytes and neurons is known as the tripartite synapse [18]. Within this fundamental unit, both glutamate release and ion buffering occur at critical sites. As TREK-1 has been identified as a participant in the rapid release of glutamate at tripartite synapses [19], its role in glia–neuron interactions is a focal point of public and scientific interest.

While the association between epileptic seizures and TREK-1 signalling is well documented [3], the precise spatiotemporal dynamics of astrocyte–neuron interactions during seizures are not yet fully understood. This review critically examines current studies focused on the function of TREK-1 and its involvement in epilepsy or seizures, with the aim of providing a more comprehensive understanding of the role of TREK-1 in epilepsy through glia‒neuron interactions. Our understanding of TREK-1 in epilepsy will be improved and provide new ideas for drug development.

## K2P and TREK-1

### Structure of TREK-1

TREK-1, a member of the two-pore domain potassium channel family that functions as a “background leakage potassium channel”, plays important roles in regulating neuronal excitability and maintaining the resting membrane potential; its functional abnormalities are closely associated with epilepsy, pain, depression and cerebral ischaemic neurological diseases [9, 20]. Mammals have 15 genes encoding the K2P family channels, which can be classified into 6 subfamilies (TWIK, TASK, TREK, THIK, TALK, and TRESK) based on their structures [3].

**Fig. 1:**
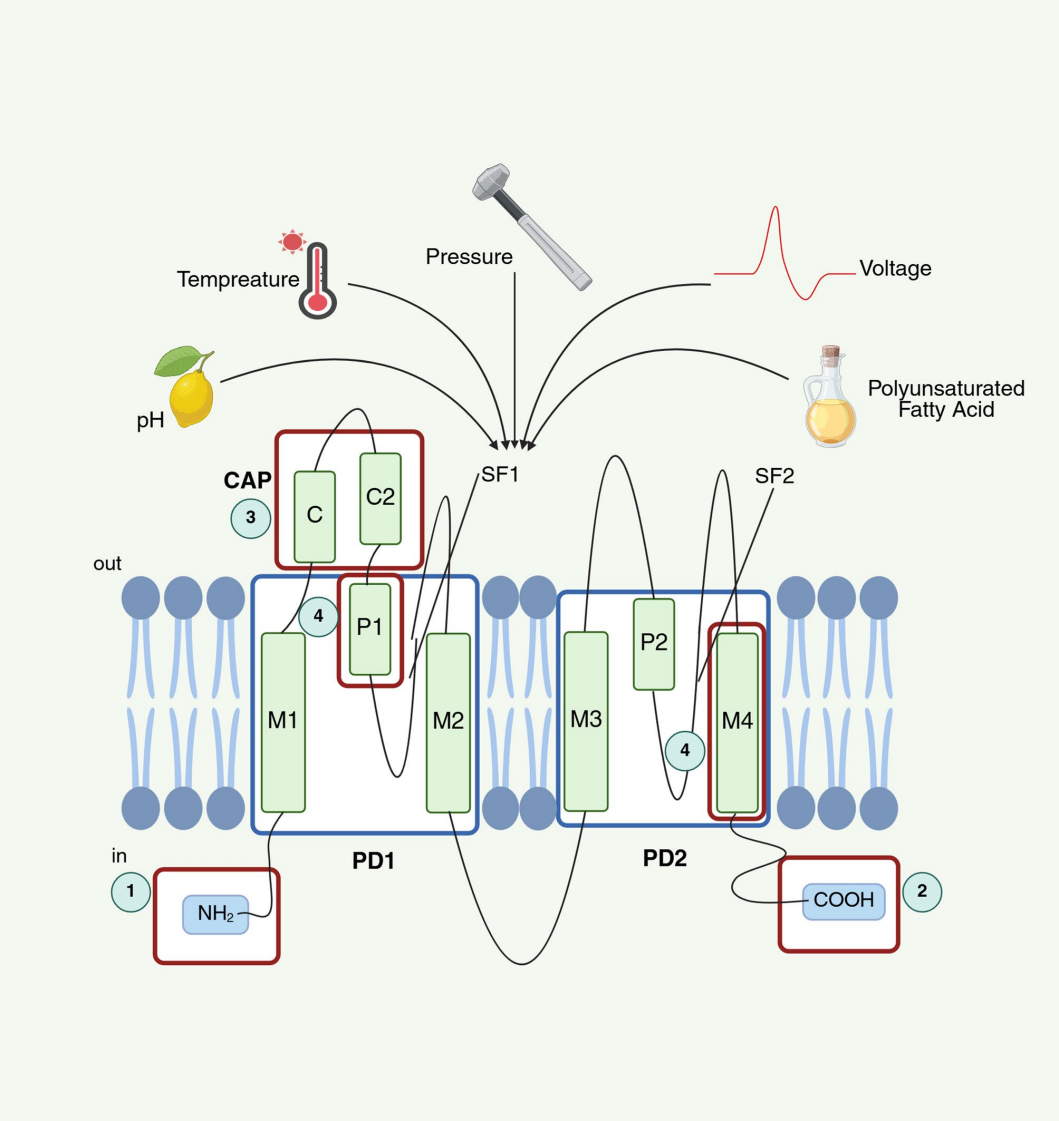
The structure and regulation of TREK-1. TREK-1 has two subunits, each with two pore domains (PD1 and PD2). Each subunit consists of two helical transmembrane domains (M1 and M2, M3 and M4), a pore helix (P1 and P2) and selectivity filters. There are four targets of possible drug action has been reported. They are ①N-terminal, ②C-terminal, ③extracellular region called “cap” and ④K2P modulator pocket. The intracellular side has a short amino terminus and a long carboxyl terminus, and the extracellular side has a loop between M1 and P1, forming a special “cap” structure on the pore with an α-helix. The N-terminus and C-terminus of the protein form intracellular structural domains, the latter being active in transmitting electrical, chemical, and mechanical signals into the channel. This extracellular structural domain, “cap”, defines two tunnel-like lateral gates as extracellular ion pathways and partially impedes the direct movement of ions into the extracellular environment. K2P molecular pocket comprises a “P1 face” and an “M4 face”. The figure was created based on Production of K2P2.1 (TREK-1) for structural studies [44]. Created with http://BioRender.com

Structurally, TREK-1 is composed of two subunits of homologous/heterologous dimers (such as with TWIK-1), and each subunit contains four transmembrane helices (M1–M4) and two pore domains [21](Fig. 1). Both pore domains contain the GFG motif (different from the common potassium channel motif GYG), which affects ion selectivity and conductivity [22]. Notably, recent attention has focused on the K2P modulation pocket and the “cap” as potential activation targets, especially based on the activation and inhibition of TREK-1 [22–24]. The intracellular structural domain comprises the N-terminal and C-terminal ends of the protein, with the C-terminal end playing a pivotal role in converting electrical, chemical, and mechanical signals into channel signals [24, 25]. Currently, TREK-1 activators reported in the literature include polyunsaturated fatty acids (PUFAs), such as arachidonic acid(AA), α-linolenic acid (ALA), cis-4,7,10,13,16,19-docosahexaenoic acid, ML-67, ML67-33, ML335 and ML402 [9, 11, 23, 26–28]. Among these molecules, the ML335 and ML402 action sites are located in the K2P modulator pocket [22]. The PUFAs, (AA, ALA), cis-4,7,10,13,16,19-docosahexaenoic acid, ML-67 and ML67-33 action sites are located in the C-terminus of TREK-1 [25, 28]. TREK-1 inhibitors reported in the literature include spadin, retroinverso analogues of spadin, fluoxetine, N-[2-[(1 S, 4 S, 5 S)-5-bicyclo [2.2.1] hept-2-enyl] ethyl]-5-[(2, 4- difluorophen-oxy) methyl]-1, 2-oxazole-3-carboxamide(SID1900), 3-(nitromethyl) isobenzofuran-1(3 H)-one(Lig4-4) and N-(4-cholorphenyl)-N-(2-(3,4-dihydrosioquinolin-2(1 H)-yl)-2-oxoethyl)methanesulfonamide(TKDC) [27, 29, 30]. Among these inhibitors, N-(4-cholorphenyl)-N-(2-(3,4-dihydrosioquinolin-2(1 H)-yl)-2-oxoethyl)methanesulfonamide(TKDC) action sites are located in the “cap” [22].

However, the specific binding sites of other activators and inhibitors need to be examined further. Specifically, the C-terminus serves as the site where cAMP mediates the inhibition of TREK-1 [31, 32]. These unique structures distinguish its regulatory mechanism from those of other K2P members. During physiological processes, members of the K2P family often form homodimers or heterodimers with other members from the same or different subfamilies to facilitate their functional roles [33]. Common heterodimer compositions in the K2P family are TWIK-1 and TREK-1 with TREK-2, as well as TRESK and TASK-5 with either TASK-1 or TASK-3 [34–36].

### Distribution, regulation and functions of TREK-1

An analysis of the distribution of TREK-1 in the human body reveals higher expression levels in the central nervous system than in peripheral tissues [37, 38]. Specifically, TREK-1 is highly expressed mainly in the olfactory bulb, hippocampus, hypothalamus, cerebral cortex, caudate nucleus and central pontine nucleus (γ-GABA amino butyric acid interneurons) in the brain [39]. In peripheral tissues, TREK-1 is abundantly expressed in gastrointestinal smooth muscle, lung tissue and vascular endothelial cells, as well as in atrial and ventricular myocytes [40]. Based on its distribution and extensive research findings, TREK-1 not only is implicated in neurological disorders but also plays critical roles in cardiovascular and pulmonary diseases. At the cellular level, TREK-1 is localized in neuronal dendrites and astrocytes within the hippocampus [19]. However, further investigations are needed to elucidate the detailed expression pattern and level of TREK-1 in astrocytes, particularly in human patient populations.

TREK-1 has been shown to be involved in the maintenance of resting membrane potential, making it relevant to anesthesia and various neurological disorders, including epilepsy [41], pain [20], depression [30] and ischemia [42]. TREK-1 regulation is modulated by a diverse array of physical and chemical factors, such as membrane stretching, changes in the membrane potential, temperature, pH, and the levels of PUFAs and phospholipids (Fig. 1) [4–8]. Furthermore, TREK-1 is regulated by second messengers linked to receptor pathways, similar to most channel proteins. Membrane depolarization activates the Gαq-coupled receptor pathway, which subsequently depletes phosphatidylinositol-4,5-bisphosphate (PIP2) through the action of phospholipase C, ultimately resulting in the inhibition of TREK-1 [25, 31, 43]. These mechanisms underline the role of TREK-1 in the regulation of neuronal excitability.

## Balance of K^+^

Epilepsy is a neurological disorder involving abnormal discharges in different parts of the brain. This occurs mainly because of synchronized neuronal firing, in which K^+^ currents are intimately involved. The regulation of the balance between the intracellular and extracellular levels of K^+^ is of critical importance to ensure the correct maintenance of the environment of the brain. In addition to the major potassium channel of astrocytes named Kir4.1, TREK-1, another potassium channel of astrocytes in the hippocampus, is closely involved in epilepsy [9, 45, 46].

In animal models, the activation of TREK-1 leads to the hyperpolarization of both presynaptic and postsynaptic membranes and subsequent neuronal inhibition [47]. Conversely, the inhibition or knockdown of the channel results in neuronal depolarization and excitation [16, 48]. Lau et al. [49, 50] investigated the role of lysophospholipids (LIN), an activator of TREK-1, and observed neuroprotective effects, i.e., a decrease in excitability, which was attributed to the hyperpolarization of the cell membrane. Additionally, a TREK-1 mutant, TREK-M, characterized by enhanced constitutive activity, was shown to suppress neuronal excitation by augmenting potassium currents and lowering the resting membrane potential [46]. Furthermore, TREK-M was shown to decrease the seizure duration and reduce neuronal death in a chronic epilepsy model [46]. The current evidence suggests a potential association between TREK-1 activation and the remission of epilepsy. In animal models, the application of TREK-M mutants or LIN reduced neuronal excitability and the seizure duration, phenomena associated with increased astrocyte potassium currents [46, 49, 50]. However, whether the activation of TREK-1 inhibits epilepsy directly through increased potassium currents still needs to be verified, and the influence of other mechanisms has not been completely excluded. Future studies need to use specific agonists of TREK-1 in conditional knockout models to clarify the causal relationship between TREK-1-mediated potassium homeostasis and antiepileptic effects (Table 1).

**Table 1:** Neuronal studies of TREK-1 in epilepsy or seizure activity

	Patients	Mice				Cell	
**Model**	Human [15] (Temporal lobe epilepsy)	TREK-1-/- [9, 45]			TREK-M [46]	CHO [43]	
**Intervention**		PUFA	KA or PTZ	Hyperbaric chamber	Lithium-pilocarpine	Caffeine	Theophylline
**Detection indicators**							
Rating scales^a^	√						
Electrophysiology						√	√
Staining^b^	√				√		
Score^c^		√	√	√	√		
% Death		√	√		√		
EEG activity		√	√				
**State of TREK-1**	Activation	Activation	Inhibition	Inhibition	Activation	Inhibition	
**Effects**	Protective	Protective	Harmful	Harmful	Protect	Harmful	

Abbreviations: CHO: Chinese hamster ovary; TREK-M: TREK-1 mutant

### Net K^+^ uptake

Astrocytes, rather than neurons, are responsible for maintaining the homeostasis of K^+^ within the synaptic cleft by facilitating the uptake of extracellular K⁺ [45]. This process is essential for preventing neuronal overexcitation and is believed to be primarily supported by Na^+^-K^+^-ATPase (NKA), which plays a significant role, alongside the Na^+^-K^+^-2Cl^−^ cotransporter (NKCC), which mitigates K^+^ currents and initiates K^+^ uptake. Additionally, inwardly rectifying potassium channel 4.1 (Kir4.1) is thought to have a substantial impact by inwardly rectifying K⁺ currents [51]. During synaptic transmission, astrocytes regulate neuronal activation by modulating [K^+^]_o_ through these mechanisms. Furthermore, astrocyte swelling has been regarded as an indicator of net K^+^ uptake [45]. A three-compartment computational model was developed in 2015 and was used to assess K^+^ dynamics among neurons, astrocytes, and extracellular interstitial spaces; the results showed that Kir4.1 is both responsible for and sufficient to mediate astrocytic depolarization and K^+^ removal from astrocyte membranes within 6–9 s following neuronal activity [52]. However, the transient swelling of astrocytes following acute neuronal activity is initiated by the uptake of potassium ions through TREK-1 rather than Kir4.1 or NKCC [53]. A previous study showed that the uptake of K^+^ was significantly inhibited following TREK-1 knockdown, whereas Kir4.1 knockdown only slightly affected the membrane potential [53]. These findings suggest that TREK-1, rather than Kir4.1, serves as the primary channel facilitating net K^+^ uptake following acute neuronal activity. This finding contradicts previous conclusions, which may be attributed to variations in the experimental setup.

During the uptake process associated with acute neuronal activity, water molecules, along with K^+^ released from N-methyl-D-aspartic acid receptors (NMDARs) and α-amino-3-hydroxy-5-methyl-4-isoxazole-propionic acid receptors (AMPARs), traverse the astrocyte-specific water channel aquaporin-4 (AQP4), resulting in a transient increase in the astrocyte volume [19, 53]. Concurrently, astrocyte swelling leads to a reduction in the synaptic cleft volume, thereby increasing the concentration of glutamate within the cleft [54]. 4-(2-Butyl-6,7-dichloro-2-cyclopentylindan-1-one-5-yl)oxybutyric acid (DCPIB), which acts as a TREK-1 activator and an AQP4 inhibitor, has been shown to increase potassium currents across astrocyte membranes [55]. This observation further implies that TREK-1 plays a crucial role in net K^+^ uptake [53]. However, validation using a TREK-1-selective agonist would provide more definitive evidence.

**Fig. 2:**
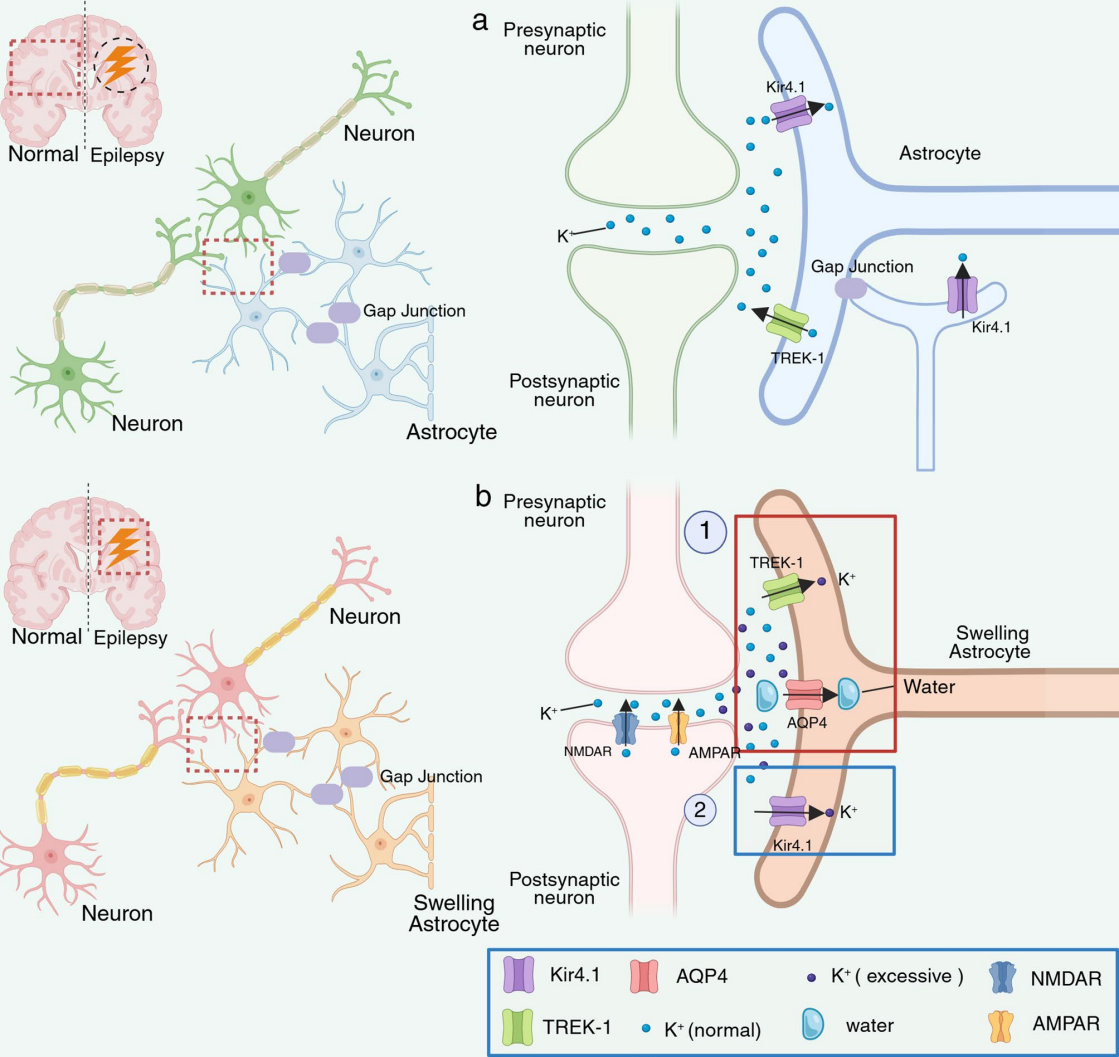
Net K^+^ uptake in normal humans and epilepsy. Normally, net K^+^ uptake is accomplished by Kir4.1, which conducts K^+^ by gap junctions to maintain K^+^ homeostasis in the brain (Panel **a**). However, instantaneous acute and intense neuronal stimulation is hypothesized to lead to astrocyte swelling in patients with epilepsy, and TREK-1 is able to conduct excess K^+^ from the synaptic cleft into astrocytes along with the transport of water molecules mediated by APQ4 (Panel **b**) [53]. Created with https://BioRender.com

In contrast to the conventional model, acute neuronal activity, which induces transient swelling, is driven primarily by TREK-1-mediated K⁺ influx rather than by Kir4.1- or NKCC1-mediated influx [54]. This discrepancy may arise from differences in the models used, as traditional studies predominantly focus on steady-state potassium cycling, which is largely governed by Kir4.1. Conversely, the onset of acute neuronal bursts of intense activity, such as seizures, relies on TREK-1-mediated K⁺ influx. This apparent paradox may indicate a temporal variation in the mechanism of net K⁺ uptake: TREK-1 may serve as a principal mediator of acute potassium uptake during intense neuronal activity, whereas Kir4.1 and NKCC1 may be responsible for maintaining basal homeostasis. The relative contributions of these mechanisms could be further elucidated using TREK-1/Kir4.1 double knockout models or time-difference experiments, which would also allow for the exploration of potential synergistic or antagonistic interactions between these pathways [53] (Fig. 2).

### TREK-1 is not the key molecule involved in the spatial buffering of K^+^

In addition to net K^+^ uptake, the maintenance of K^+^ homeostasis relies on spatial buffering mechanisms, the latter of which disperses local K^+^ accumulation through glial cell networks. The spatial buffering of K^+^ occurs in individual astrocytes or in astrocytes via Connexin43 (CX43) and Connexin30 (CX30) gap junctions. Gap junctions play a key role in the spatial buffering of K^+^, which is involved in maintaining the homeostasis of K^+^ in the brain [45]. In 2014, Larsen and Macaulay hypothesized that Kir4.1 is largely responsible for mediating the spatial buffering of K^+^ in astrocytes [56]. A three-compartment computational model assessing K^+^ dynamics between neurons, astrocytes, and extracellular interstitial spaces indicated that Kir4.1 is responsible for and sufficient to mediate depolarization in astrocytes and K^+^ removal from astrocyte membranes within 6–9 s after neuronal activity [52]. However, the contribution of Kir4.1 to the spatial buffering of K^+^ is unclear, as some reports have failed to obtain meaningful evidence for the role of Kir4.1 in K^+^ clearance in different brain regions. However, TREK-1 deficiency does not alter the conductance or network function of astrocyte membranes, suggesting that TREK-1 may not be involved in the spatial buffering of K^+^ through gap junctions [57].

### Passive conductance mediated by the TWIK-1/TREK-1 heterodimer

Mature astrocytes display a highly negative and stable membrane potential, a feature known as “astrocyte passive conductance”, which is associated with K^+^ buffering [45, 58]. Unlike conventional voltage gating, this membrane potential usually has a linear current‒voltage relationship, indicating the maturation of astrocytes [58]. As a method to identify the exact molecule involved in passive conductance, Bae [59] et al. used spadin, a specific blocker of TREK-1, to reduce TREK-1-mediated K^+^ currents in cultured astrocytes and modulate astrocyte passive conductance in hippocampal slices. Furthermore, silencing TWIK-1 by shRNA interference resulted in reduced passive conductance in cultured astrocytes. This finding implies that the heterodimer formed by TWIK-1 and TREK-1 is responsible for passive conductance [34, 59]. The passive conductance of astrocytes plays an essential role in epilepsy [58, 60]. Bae focused on Na^+^/H^+^ exchange regulator-1 (NHERF-1), which is able to bind to TWIK-1/TREK-1 in cultured hippocampal astrocytes. NHERF-1 overexpression inhibited the activity of TWIK-1/TREK-1 [61]. More importantly, the overexpression of NHERF-1 also resulted in decreased passive conductance of astrocytes and reduced TWIK-1/TREK-1-mediated K^+^ efflux, leading to the depolarization of the membrane potential and increased susceptibility to seizures induced by kainic acid (KA) [61]. This evidence suggests that TREK-1 is a key target that regulates passive conductance in astrocytes and plays an important role in epilepsy. Notably, the target of action of NHERF-1 is located at TREK-1 N4 (a part of the N-terminus of TREK-1), which can restore astrocyte passive conductance that is reduced when NHERF-1 is overexpressed and significantly attenuates seizure behaviour induced by KA [61]. Therefore, an excessive focus on any channel while completely ignoring the contribution of other channels to passive conductance should be avoided; more importantly, a global view of the effect of the TWIK-1/TREK-1 heterodimer on the passive conductance of astrocytes is needed [62].

Using hippocampal sections from rats, Zhou [58] demonstrated that TWIK-1 and TREK-1 act on passive conductance separately. However, Hwang [34] introduced a TWIK-1 shRNA or TREK-1 shRNA into mice using a lentivirus and found that the TREK-1/TWIK-1 heterodimer is a key molecule in astrocytes. In addition, Du et al. [63] utilized knockout mice and crossed TREK-1^−/−^ and TWIK-1^−/−^ mice to obtain mice in which both channels were knocked out, and the results were inconsistent with those of previous studies. This discrepancy may stem from a fundamental difference in experimental models. One potential explanation for this phenomenon is that the gene knockout model employed by Du et al. exhibits a complete absence of TREK-1 or TWIK-1 at birth. This could result in astrocytes assuming a compensatory role by regulating alternative ion channels during development, thereby overshadowing the physiological function of TWIK-1/TREK-1. The long-term compensatory effect maintains passive conductance normally in equilibrium but does not reflect the acute function of the target gene. Hwang et al. [34]. used a viral interference model in adult mice to acutely inhibit TREK-1, which targets astrocytes directly. This approach can avoid the activation of developmental compensatory mechanisms and reveal a direct regulatory effect of the TWIK-1/TREK-1 heterodimer on passive conductance. This model more closely mimics the acute ionic imbalance during seizures. This compensatory mechanism may be the main reason for the difference. Thus, given the importance of TWIK-1/TREK-1 in epilepsy, the mechanisms by which it affects passive conductance need to be studied in depth (Table 2).

**Table 2:** Roles of TREK-1 and Kir4.1 in astrocytic K⁺ clearance

Category	TREK-1	Kir4.1
Gene name	KCNK2	KCNJ10
Channel type	Two-pore domain K⁺ channel (K2P family)	Inwardly rectifying K⁺ channel (Kir family)
Astrocytic localization	The surface of the cell body and processes (nonmicrodomain) [19]	Distal endfeet (perivascular and perisynaptic microdomains) [64]
Primary function	Passive conductance: Mediates background K⁺ currents, stabilizes the resting membrane potential [65]	Spatial buffering: Facilitates K⁺ redistribution via gap junctions (CX43/CX30) [66]
Key molecular partners	TWIK-1 (forms heterodimer) [58] NHERF-1 (inhibits channel activity) [61]	AQP4 (colocalized at endfeet) NKCC1 (cooperates in K⁺ uptake) [56,67]
Functional evidence	Supporting:	Supporting:
	The TREK-1/TWIK-1 heterodimer mediates passive conductance in hippocampal astrocytes [34,62]	Kir4.1 knockdown impairs K⁺ uptake and extracellular K⁺ clearance [68]
	A TREK-1-specific blocker (spadin) reduced passive conductance [59]	Conditional Kir4.1 knock-out mice show impaired K⁺ buffering [69]
	NHERF-1 overexpression inhibits TREK-1 activity, increasing seizure susceptibility [70]	Kir4.1/NKCC1 synergistically regulate K⁺ uptake in tripartite synapse models [56]
Controversial evidence	Negative:	Negative:
	TREK-1 KO mice show no baseline electrophysiological changes in astrocytes [63]	Kir4.1-independent K⁺ spatial buffering is observed in the optic nerve [69]
	TWIK-1/TREK-1 double KO does not alter passive conductance in situ [62,67]	
Pathological relevance	Acute K⁺ buffering during acute neuronal activity	Maintains baseline K⁺ homeostasis under physiological conditions
	Linked to epileptogenesis via the NHERF-1–TREK-1 interaction	The loss of Kir4.1 exacerbates epilepsy and synaptic dysfunction
Therapeutic potential	TREK-1 activators may reduce excitotoxicity	Kir4.1 enhancers are proposed to treat epilepsy

## TREK-1 and GLT-1 modulate glutamate homeostasis in epilepsy

Glutamate is a neurotransmitter that regulates neuronal excitability. Excessive glutamate can lead to excitotoxicity, resulting in cell death [71, 72]. Glutamate contributes to the pathogenesis of epilepsy primarily through an imbalance in the glutamate‒glutamine cycle within the brains of individuals with epilepsy. This imbalance is characterized by (1) increased extracellular concentrations of glutamate, (2) a reduction in cytosolic glutamate dehydrogenase levels within astrocytes, and (3) modifications in the activities of glutaminase and glutamate dehydrogenase enzymes [72]. As a critical enzyme for glutamate and ammonia detoxification in brain tissue, glutamine synthetase is part of the glutamate‒glutamine cycle and altered during mesial temporal lobe epilepsy [72]. Astrocytes are crucial in regulating extracellular glutamate levels, thereby playing a significant role in maintaining synaptic homeostasis at tripartite synapses [73–75]. In TREK-1 knockout mice, a marked reduction in the inhibition of glutamate release from nerve terminals in the cerebral cortex by volatile anaesthetics was observed [76]. These findings suggest that the role of TREK-1 in epilepsy is linked to the regulation of glutamate release within the neural network [19, 34]. Further investigations into the involvement of TREK-1 in astrocyte–neuron interactions in the regulation of glutamate are warranted.

**Fig. 3:**
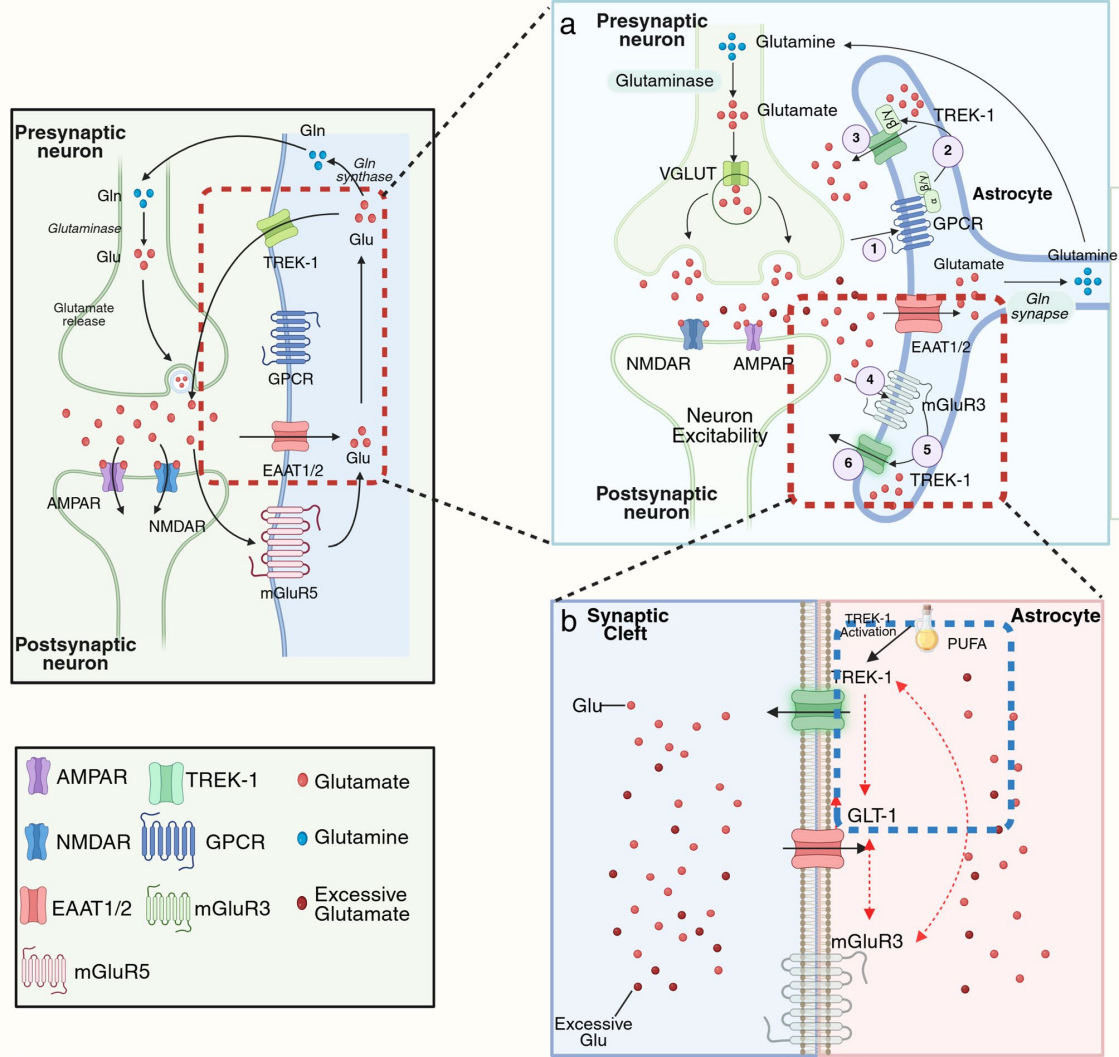
TREK-1 plays a role in glutamate regulation. The activation of the GPCR by a GPCR agonist results in the dissociation of Gβγ, which binds to TREK-1 and becomes a channel that mediates the rapid release of glutamate. Similarly, mGluR3 rapidly releases glutamate. PUFAs, as TREK-1 activators, are thought to exert neuroprotective effects, such as on epilepsy. ①GPCR is activated by GPCR agonist. ②Gβγ binds to TREK-1, turning it into a glutamate-releasing channel; ③Rapidly release of glutamate from astrocytes into the synaptic gap via TREK-1; ④Glutamate released from the presynaptic membrane is able to stimulate mGluR3 on the astrocyte membrane; ⑤Gβγ also binds to TREK-1, turning it into a glutamate-releasing channel; ⑥TREK-1 also involved in glutamate release quickly (Fig. **3a**). PUFAs are presumed to act by activating TREK-1 and then directly or indirectly upregulating GLT-1 expression, thereby exerting a protective effect. When excess extracellular glutamate is present, GLT-1 transports excess glutamate into astrocytes. TREK-1, GLT-1, and mGluR3 may interact and cooperate to regulate glutamate (Fig. **3b**). Created with https://BioRender.com

Glutamate release from astrocytes has been shown to induce transient depolarization and seizure-like discharges in CA1 pyramidal neurons [77]. The release of glutamate from astrocytes is significantly reduced after the expression of the TREK-1 shRNA, suggesting that this channel not only contributes to but also directly mediates the process [34]. The specific mechanism underlying this release is illustrated in Fig. 3a and primarily involves the activation of G protein-coupled receptors (GPCRs). Upon activation by specific agents, astrocytes release glutamate through the TREK-1 channel [19]. Activators of Gi-protein-coupled receptor G_i_-GPCRs (baclofen, a GABA_β_ agonist; the GABA_β_ agonist 2-chloro-N(6)-cyclopentyladenosine; and the cannabinoid 1 receptor agonist and adenosine receptor A1 agonist arachidonyl-2’-chloroethylamide) facilitate the activation of G_i_-GPCRs (GABAβ, cannabinoid 1 receptor, GABAβ, adenosine receptor A1, cannabinoid 1 receptor, and µ-opioid receptor). Additionally, TREK-1 interacts with dissociated G_βγ_ subunits [19], where the C-terminus of the G_γ_ subunit (GNG4) binds to the N-terminus of TREK-1, resulting in the opening of the glutamate-permeable TREK-1 channel [34]. Furthermore, subsequent experiments revealed that the specific molecule that interacts with Gβγ is TWIK-1/TREK-1, with the binding site located at the C-terminus of TREK-1 [34, 78].

Previous research has shown the extracellular glutamate concentration in the hippocampus increase during seizures and following the cessation of epilepsy in the human brain [79]. Elevated extracellular glutamate levels may result from excessive glutamate release or reduced reuptake [74]. Notably, glutamate transporter protein-1 (GLT-1) plays a vital role in maintaining low extracellular glutamate concentrations in the synaptic cleft, as GLT-1 is responsible for 80–90% of glutamate clearance in the CNS [80]. GLT-1 is expressed predominantly in astrocytes, with only 5–10% expressed in neurons [81]. A reduction in the level of GLT-1 adversely affects the ability of astrocytes to clear glutamate, thereby exacerbating the imbalance of extracellular glutamate in individuals with epilepsy [82]. GLT-1 acts as a glutamate transporter channel, and abnormalities in GLT-1 lead to abnormal glutamate efflux, triggering neuronal excitotoxicity and seizures [83]. GLT-1 knockout mice exhibited fatal spontaneous epilepsy, whereas the overexpression of GLT-1 mitigated the recurrent seizures induced by pilocarpine and prevented seizure-induced neuronal death in GLT-1 transgenic mice [84]. Previous studies have shown that the level of GLT-1 protein is downregulated during the development of epilepsy [85]. Similarly, a decrease in GLT-1 levels after an initial increase was observed in a mouse model subjected to an intrahippocampal KA injection [86]. Moreover, the degree of dysfunction caused by abnormal mutations in GLT-1 is directly and positively correlated with the clinical severity of epilepsy [87]. Mechanisms proposed to address the lower expression of GLT-1 or mutations in GLT-1 may represent a novel strategy for the treatment of drug-resistant epilepsy; however, the underlying molecular cascade of responses remains largely unexplored. According to previous reports, TREK-1 may play a neuroprotective role during epileptic states [9]. Nonetheless, this effect may be limited and insufficient to fully restore normal neuronal function. There is no direct evidence linking TREK-1 and GLT-1(EATT2), but multi-disease models indicate that there may be a synergistic effect in regulating glutamate homeostasis. In Alzheimer’s disease (AD) models, alpha-linolenic acid (ALA), an activator of TREK-1, has been shown to upregulate astrocytic GLT-1 expression, which can reduce the accumulation of glutamate and prevent it from exerting its effects, resulting in the relief from neurotoxicity [11]. In cerebral ischemia studies, activating TREK-1 by arachidonic acid (AA) could maintain sufficient electrochemical gradients to drive the glutamate uptake function of GLT-1 [88, 89], while inhibiting of TREK-1 activity with quinine could suppress the uptake of glutamate by astrocytes under hypoxia conditions [89]. Based on the above reports, we speculated that TREK-1 might regulate glutamate homeostasis in epilepsy by interacting with GLT-1, that is, TREK-1 activation indirectly upregulated the expression or function of GLT-1. This regulation promoted glutamate transport from the synaptic gap to astrocytes, thereby alleviating glutamate accumulation in the synaptic gap and contributing to neuroprotection. Furthermore, the polyunsaturated fatty acids (PUFAs), including AA and ALA, might ultimately exert neuroprotective effects by activating astrocyte TREK-1, which in turn facilitates the glutamate scavenging capacity of GLT-1 [11, 88, 89]. However, the specific mechanism of the TREK-1-GLT-1 signaling pathway still needs to be further verified.

Besides metabotropic glutamate receptor 5, group II metabotropic glutamate receptor 3 (mGluR3) is a metabotropic glutamate receptor expressed on astrocytes and can sense the concentration of glutamate in the synaptic cleft [90–92]. mGluR3 is able to activate TREK-1 through G protein signalling, which mediates the rapid release of glutamate [93]. The activated PAR1 receptor signals through mGluR3, which prompts TREK-1 to open and mediate the rapid release of glutamate, resulting in a transient increase in the concentration of glutamate in the synaptic cleft [93]. Although TREK-1 expression is low in astrocytes, its aggregation in microstructural domains may amplify its release effect [19]. Paradoxically, the sustained activation of mGluR3 enhances glutamate clearance by increasing GLT-1 and GLAST expression. In enriched human cultured astrocyte models, mGluR3 agonists significantly increase the levels of GLT-1 and attenuate glutamate excitotoxicity [94]. mGluR3 expression is upregulated in TLE rat models and patients and may exert neuroprotective effects through GLT-1-dependent reuptake [94, 95]. TREK-1 activators, such as α-linolenic acid or arachidonic acid, concomitantly upregulate the expression of GLT-1 and reduce the accumulation of glutamate. These findings suggest that the two proteins may share upstream regulatory pathways. Contradictory results concerning the expression of mGluR3 in TLE patients have been reported, which may reflect pathological stage- or brain region-specific regulation [96]. No direct evidence for the regulation of GLT-1 by mGluR3 is available, but the above evidence indicates that the three are inextricably linked; experimental validation of the colocalization or signalling pathways is necessary to verify the three relationships. The possible spatiotemporal specificity of the interactions among the three proteins still needs to be experimentally confirmed (Fig. 3).

## Conclusions

The dysregulation of K^+^ and glutamate release is implicated in the abnormal excitability of neurons, leading to epileptic seizures. The underlying link between the TREK-1 channel and epilepsy, as well as its associated comorbidities, has already been well established. Given the intrinsic relationship between the K2P channel and glutamate release, TREK-1 has been identified as an important regulator of both the buffering of K^+^ and the circulation and release of glutamate in astrocytes. TREK-1 influences the “epileptogenic storm” by influencing neuronal excitability and glutamate release; furthermore, it plays a role in modulating the astrocyte volume, positioning it as a potential therapeutic target within astrocytes. Nevertheless, the specific mechanisms by which TREK-1 modulates glutamate and K^+^ currents in the context of epilepsy require further investigation. Understanding the regulation of TREK-1 in relation to the astrocyte volume is crucial for elucidating the pathophysiology of CNS diseases. Importantly, this review specifically addresses the interaction between TREK-1 and astrocytes, but these factors are not the sole contributors to epileptogenesis. Further studies are needed to explore additional mechanisms involved in this complex process.

## Data Availability

No datasets were generated or analysed during the current study.

## Abbreviations

AA: Arachidonic acid
ALA: α-linolenic acid
AMPAR: α-amino-3-hydroxy-5-methyl-4-isoxazole-propionicacid receptor
AQP4: Aquaporin-4
cAMP/PKA: Cyclic adenosine monophosphate/ protein kinase A
CNS: Central nervous system
DCPIB: Derivative-4-(2-butyl-6,7-dichlor-2-cyclopentylindan-1-on-5-yl) oxobutyric acid
GLT-1: Glutamate transporter protein-1
GPCR: G protein-coupled receptors
KA: Kainic acid
Kir4.1: Inwardly rectifying potassium channel 4.1
K2P: Two-pore domain potassium
Lig4-4: 3-(nitromethyl) isobenzofuran-1(3 H)-one
LIN: Lysophospholipids
mGluR3: Metabotropic glutamate receptor-3
NHERF-1: Na^+^/H^+^ exchange regulator-1
NKA: Na^+^-K^+^-ATPase
NKCC: Na^+^-K^+^-2Cl^−^ cotransporter
NMDAR: N-methyl-D-aspartic acid receptor
PIP2: Phosphatidylinositol-4,5-bisphosphate
PTZ: Pentylenetetrazol
PTZ: Pentylenetetrazol
PUFA: Polyunsaturated fatty acids
SID1900: N-[2-[(1 S, 4 S, 5 S)-5-bicyclo [2.2.1] hept-2-enyl] ethyl]-5-[(2, 4- difluorophen-oxy) methyl]-1, 2-oxazole-3-carboxamide
TALK: TWIK-related alkaline activated K^+^ channel
TASK: TWIK-related, acid-sensitive K^+^ channel
THIK: Tandem pore domain halothane-inhibited K channel
TKDC: N-(4-cholorphenyl)-N-(2-(3,4-dihydrosioquinolin-2(1 H)-yl)-2-oxoethyl)methanesulfonamide
TRAAK: TWICK-related arachidonic acid-activated K^+^ channel
TREK: The TWIK-related K^+^ channel
TRESK: TWIK-related spinal cord K^+^ channel
TWIK: Two-pore domain weakly inward rectifying K^+^ channel
TLE: Temporal lobe epilepsy
